# Ethical Design and Use of Robotic Care of the Elderly

**DOI:** 10.1007/s11673-022-10181-z

**Published:** 2022-03-21

**Authors:** Carolyn Johnston

**Affiliations:** Law School, University of Tasmania, Sandy Bay, Hobart, TAS 7005 Australia

**Keywords:** Robotic care, ethics, ethics by design, ethics committees

## Abstract

The Australian Royal Commission into Aged Care Quality and Safety acknowledged understaffing and substandard care in residential aged care and home care services, and recommendations were made that that the Australian Government should promote assistive technology within aged care. Robotic care assistants can provide care and companionship for the elderly—both in their own homes and within health and aged care institutions. Although more research is required into their use, studies indicate benefits, including enabling the elderly to live independently at home, assistance with medication and monitoring of safety. Nevertheless, there are inherent ethical challenges in the use of robots as carers, including loss of privacy, unwarranted restrictions on autonomy, lack of dignity, deception, and the exacerbation of loneliness. Ethics by design can counter these issues in development of robotics and clinical ethics committees have been put forward as a way of dealing with the ethical use of robotic care in healthcare institutions. In this paper I outline the ethical challenges of robotic care assistants and how these may be mediated in their design and use.

## Introduction

The increasing elderly population around the world and demand for resources to care for them is putting pressure on health and aged care providers. The number of people aged sixty and older is projected to grow to nearly 2.1 billion by 2050, with most of the increase in developing countries (World Health Organization 2021). In Australia there are approximately 4.2 million people aged sixty-five and over, 16 percent of Australia’s total population (Australian Institute of Health and Welfare 2021).

The COVID-19 pandemic has highlighted the limits of aged care institutions and, with international and state and territory borders closed, families were also prevented from caring for their elderly loved ones. Governments around the world are grappling with the viability of robotic care assistants and robotic companion pets to supplement human care and offer an antidote to loneliness. They are considering how the benefits of robotic technology can be harnessed whilst addressing inherent challenges such as data security and user safety. In its Final Report (2021) the Royal Commission into Aged Care Quality and Safety recommended that the Australian Government should use assistive technologies within the aged care programme to enable safe, independent living at home, and in the United Kingdom the government has invested thirty-four million GBP in the development of care robots to be used in the adult social care sector. *Paro*, a robotic companion pet, has been approved as a therapeutic medical device by the Food and Drug Administration in the United States.

However, concerns about the privacy, dignity, and autonomy of the elderly user demand the ethical development and function of robotic care assistants. What are appropriate protections for the use, storage, and sharing of personal data collected by a robot monitoring movements in the home of an elderly person? Should a robotic carer override the autonomy of the user to prevent harm? Is it even acceptable for robotic care assistants to replace human carers if this exacerbates loneliness and impacts on the dignity of the elderly user? Ethics by design and institutional robot ethics committees have been proposed as means to ensure that the robot’s actions remain within moral bounds and this article explores these concepts and considers their effectiveness.

## Forms of Robotic Care

Robots are well established in surgery and their use in other forms of healthcare and in adult social care has proliferated in recent years. During the COVID-19 pandemic robots proved useful in monitoring for raised temperatures, indicating presence of the virus, and disinfecting hospitals and care homes, without the risk to humans. Technology such as wearable alarms to summon help, automated pillboxes to manage medication and fall sensors already exists to assist older people to live at home. Robotic care assistants are being developed to provide companionship and assistance to the elderly and come in a variety of forms—humanoid robots which have facial features and may simulate human behaviour and robots resembling animals, such as *Paro*, the baby seal, a companion robotic pet, originally designed for use in Japanese care homes. Care robots such as the *Care O Bot* and *Henry* assist the elderly with tasks such as fetching household items and give reminders for medication and some care robots such as RIBA II can lift and manoeuvre the user.

While robots are not susceptible to the tiredness, distractibility, irritability, and limits on availability that human carers may demonstrate, they lack emotional engagement and human values, and this is a challenge to the appropriate use of robotic technology in health and social care (Moyle 2019). How can robots be designed and used in a way that is ethically acceptable and promotes the welfare of the user? In 2019 the CSIRO published a Discussion Paper which considered how existing laws and ethical principles could be applied in the context of Artificial Intelligence which drives robotic care (Dawson et al. 2019). The core principles identified include generation of net-benefits, no harm or deception to the user and privacy protections for the data collected and used.

The literature identifies key ethical challenges in the use of robotic care assistants - autonomy, privacy, dignity, and bias (European Parliament 2017; Houses of Parliament, Parliamentary Office of Science and Technology 2018). Where a robot is used in the home of an elderly person there is intrinsic interference with the privacy of the user, as the robot collects and stores data and some robots even have cameras which “see” into the user’s personal domain, monitoring how the older person moves around the home which may trigger intervention and also capture movements of visitors. Autonomy of the user may be suppressed where the robot provides medications to a timetable or even overridden, for example where the elderly person is prevented from climbing on a chair to reach something in a cupboard. Does the outcome of the decision of the robot generate a net benefit? A fall is prevented but at a cost to the autonomous choice and dignity of the individual.

Legislation addresses some aspects of robotic care. The Privacy Act 1988 (Cth) deals with data privacy, although how this is navigated in AI may prove difficult. The 2020 review of the Act recognized that more personal information about individuals is being captured through artificial intelligence and any changes to the legislation should ensure that it is fit for purpose with respect to this technology. The Therapeutic Goods Act 1989 regulates medical devices, and a robotic care assistant could meet the definition of a medical device if, for example, it assists with functional disability or communication for users with dementia (Moyle 2019). Advanced robots and algorithms might be classified differently from traditional consumer products and therefore use of consumer law may require “differentiated treatment” and responsibility to regulate the safety of robotic care assistants (Magrani 2019, 11).

In addition to the challenges of applying broad regulatory principles to the complexities of AI, legislation fails to address the nuance and context of use of robotic carers, and fast paced developments in technology demands a more responsive approach. Iphofen and Kritikos argue that “policies and regulations will fail if no account is taken of the ethics of robotics” (Iphofen and Kritikos 2021, 170). “Roboethics” can be addressed both in the design of the AI system and in its application in practice.

## Ethics by Design

How can robots be designed to ensure that they make ethical decisions which are beneficial and do not inappropriately override the autonomy of elderly, vulnerable individuals who use them? Ethics by design describes the,

… methods, algorithms and tools needed to endow autonomous agents with the capability to reason about the ethical aspects of their decisions, and the methods, tools and formalisms to guarantee that an agent’s behavior remains within given moral bounds. (Dignum et al. 2018, 61)

Ethical design of AI systems integrates human values such as welfare of the elderly, dignity, and respect for autonomy. But robots are human creations and act within bounded categories and the challenge for researchers and developers of AI is development of algorithms which can “discern” between ethical and unethical options and then translate human values into technical requirements. Blake proposes teaching ethics to AI designers who can then “teach” ethics to a machine (Blake 2020). However as human moral agents, designers will respond to different scenarios according to their own priorities and values. It has been suggested that inherent biases can be counteracted using an iterative approach in design, taking account of multi stakeholder perspectives, including those of the elderly (Dignam et al. 2018).

A value sensitive approach to design of robotic carers can capture the benefits of the technology, with the welfare of the user at the core. However ethical issues in the use of robotic carers cannot be adequately “designed in,” at least not until sufficient machine learning has taken place in response to scenarios. Social interaction and connection with other humans have measurable benefits on well-being, so if robotic care replaces rather than supports human care their use may be unethical. Appropriate use of robotic carers in a health or aged care institution (rather than in the person’s own home) could be evaluated by an ethics committee.

## Ethical Use of Robotic Carers—Robot Ethics Committees

Clinical ethics committees, or groups, provide decision-making support to healthcare providers in ethically challenging cases through clinical case discussion. They can also be involved in ethical input into hospital policies. Clinical ethics committees have proliferated internationally, and there are a number of clinical ethics committees, or groups, in Australia, although the exact numbers are unknown. Blake proposes “robot ethics committees” either specially constituted or a subcommittee of a clinical ethics service, as a way to monitor ethical use of robot carers at healthcare institutions and to safeguard patients against harms arising from their use (Blake 2020). The robot ethics committee could create hospital policies and procedures that must be followed and also provide a consultation service so that ethics advice is given in response to particular clinical situations, such as use of robotic assistants where the elderly person lacks capacity to consent, use of resources to purchase robotic carers, impact on staffing, and whether the elderly person receives overall benefit.

Such ethics committees enable a soft form of governance, however they are necessarily limited to institutions that already have such an ethics service or are willing to establish one. There may be some reluctance to use yet another committee as a means to consider whether robotic aspects of care are ethically sensitive, where close attention to the human element of care may suffice. When robotic care assistants become more affordable and are purchased in greater numbers for use in private homes, the need for oversight and regulation becomes more pressing.

## Informed Consent

Informed consent provides the elderly user with a voice as to if, and how, a robotic care assistant can support them. If used in the home, then whether they are willing to accept some invasion of privacy in return for the benefits of companionship and assistance with personal care and daily tasks, or how a patient wishes care robots to be used in the course of treatment at hospital. There are concerns to be addressed as this technology develops. Given the complexity of information to be disclosed, the potential for coercion by family members who may suggest robot care to relieve them of the burden of care, and concerns around the cognitive ability of the older person to continue to exercise a choice to have robotic care, informed consent appears illusory.

## Conclusion

AI will increasingly influence and have an impact on the way vulnerable members of our society, including the elderly, are cared for in their homes and in health and aged care institutions. Government endorsed regulation by way of legislation, such as privacy and consumer protection, has the advantage of ensuring compliance through the imposition of penalties but self-regulation of AI developers has been the usual model for regulating robotics. This has been criticized as inadequate to protect the safety of the vulnerable and promote their interests, and there is scant accountability for non-adherence to self-imposed rules and standards. A report of the European Parliament Committee on Legal Affairs (2017) recommends a voluntary ethical code of conduct for robot manufacturers to guide safety and there is a role for ethics committees to develop codes of ethics for use of care robots in healthcare institutions, where such an ethics service is established. Although, due to technological limitations, ubiquitous use of robots to assist in care of the elderly in their own homes is a way off, now is the time to anticipate legal, ethical, and social challenges that they present. Robot carers can empower the elderly to lead more independent lives and to be supported to stay in their homes but at a cost of personal surveillance and a potential reduction in human interaction. Fundamentally the ethical design of machines will ensure that they are developed and used responsibility so that they benefit the elderly and are trusted to augment human care.

## References

[CR1] Australian Institute for Health and Welfare. 2021. Older Australians [website]. *Australian Institute for Health and Welfare*, November 18. https://www.aihw.gov.au/reports/older-people/older-australia-at-a-glance/contents/summary. Accessed December 23, 2021.

[CR2] Blake, V.K. 2020. Regulating care robots. *Temple Law Review* 92(3): 551-594.

[CR3] Dawson, D., Schleiger, E. Horton, J. et al. 2019. Artificial intelligence: Australia’s ethics framework—A discussion paper. Commonwealth Scientific and Industrial Research Organisation. https://apo.org.au/node/229596. Accessed December 16, 2021.

[CR4] Dignum, V., M. Baldoni, C. Baroglio, et al. 2018. Ethics by design: Necessity or curse? *AIES '18: Proceedings of the 2018 AAAI/ACM Conference on AI, Ethics, and Society*, 60– 66. 10.1145/3278721.3278745

[CR5] European Parliament. 2017. REPORT with recommendations to the Commission on Civil Law Rules on Robotics (2015/2103(INL)). https://www.europarl.europa.eu/doceo/document/A-8-2017-0005_EN.pdf. Accessed December 23, 2021.

[CR6] Houses of Parliament, Parliamentary Office of Science and Technology (2018). Robotics in social care. POSTNOTE.

[CR7] Iphofen, R., and M. Kritikos. 2021. Regulating artificial intelligence and robotics: Ethics by design in a digital society. *Journal of the Academy of Social Science* 16(2): 170-184.

[CR8] Magrani, E. 2019. New Perspectives on ethics and the laws of artificial intelligence. *Internet Policy Review* 8(3): 10.14763/2019.3.1420.

[CR9] Moyle, W. 2019. The promise of technology in the future of dementia care. *National Review of Neurology* 15: 353–359. 10.1038/s41582-019-0188-y 31073242

[CR10] World Health Organization. 2021. Ageing [website]. *World Health Organization*. https://www.who.int/health-topics/ageing#tab=tab_1. Accessed December 23, 2021.

